# Vision In SyndrOmic Neurodevelopmental disorders-15q (VISiON-15q) study: eye tracking to supplement clinical examination for detection of cerebral/cortical visual impairment in children with Angelman and Duplication of 15q Syndromes

**DOI:** 10.3389/fnhum.2026.1953706

**Published:** 2026-09-29

**Authors:** Melinda Y. Chang, Sydney Jacobs, Katherine Granger, Anna Achenbach, Charlotte DiStefano, Shafali S. Jeste

**Affiliations:** 1 Division of Ophthalmology, Department of Surgery, Children’s Hospital Los Angeles, Los Angeles, CA, United States; 2 Department of Ophthalmology, University of Southern California, Los Angeles, CA, United States; 3 Department of Pediatrics, Children’s Hospital Los Angeles, Los Angeles, CA, United States; 4 Department of Psychiatry, University of Southern California, Los Angeles, CA, United States; 5 Department of Pediatrics, University of California, Los Angeles, Los Angeles, CA, United States

**Keywords:** Angelman syndrome, cerebral visual impairment, cortical visual impairment, Dup15q syndrome, duplication of 15q syndrome, eye tracking

## Abstract

**Purpose:**

To identify children with Angelman syndrome (AS) and Duplication of 15q (Dup15q) syndrome who have cerebral/cortical visual impairment (CVI) using clinical examination combined with eye tracking.

**Design:**

Prospective cohort study.

**Participants:**

Twenty-five children (12 months to 12 years old) with AS and Dup15q syndrome, collectively termed 15q disorders.

**Methods:**

All participants underwent complete pediatric neuro-ophthalmologic evaluation, including assessment of visual acuity using the Visual Behavior Scale (VBS) and Teller acuity cards. The Higher Visual Function Question Inventory (HVFQI) was administered to assess parental report of behaviors suggestive of CVI. Eye tracking was performed in participants who were able to complete accurate calibration. Participants were also administered the Mullen Scales of Early Learning (MSEL) Visual Reception scale, as well as non-visual subscales. Parents completed the Vineland Adaptive Behavior Scales, 3^rd^ ed. (VABS-III) and Children’s Visual Function Questionnaire (CVFQ) to measure adaptive function and vision-specific quality of life, respectively.

**Main outcome measures:**

Proportion of participants classified as having diagnosis or high suspicion of CVI (15q-CVI-DHS) based on multimodal visual assessments; association between CVI status and MSEL, VABS-III, and CVFQ scores.

**Results:**

15 of 25 (60%) of 15q participants were classified as 15q-CVI-DHS. Of these, 6 (40%) were classified based on eye tracking alone. Compared to children with low suspicion of CVI, children in the 15q-CVI-DHS group had lower MSEL visual reception (17.6 ± 12.4 vs. 43.9 ± 25.7, *p* = 0.024), fine motor (21.3 ± 14.1 vs. 38.6 ± 20.6, *p* = 0.044), and verbal (15.0 ± 13.6 vs. 38.8 ± 28.0, *p* = 0.035) developmental quotients. Children in the 15q-CVI-DHS group also had lower CVFQ scores (overall 0.60 ± 0.18 vs. 0.82 ± 0.10, *p* = 0.022), without significant differences in VABS-III composite or domain scores.

**Discussion:**

Children with 15q disorders had a high rate of CVI or CVI suspect diagnosis, which was detected by eye tracking alone in 40% of cases. CVI was associated with worse vision-specific quality of life and lower developmental scores on multiple non-visual domains in these children. Future studies are needed to substantiate these findings in a larger cohort and characterize differences between genetic subtypes.

## 1 Introduction

Cerebral/cortical visual impairment (CVI) is defined as “a neurodevelopmental disorder characterized by deficits of visual function and functional vision that are caused by neurologic damage to visual pathways and processing areas in the brain” (Chang et al., 2024). CVI is a leading cause of pediatric visual impairment, yet it is suspected to be underdiagnosed, particularly in children with neurodevelopmental delays and neurogenetic disorders (Williams et al., 2021; Musleh et al., 2024). In particular, children with disorders of chromosome 15q are suspected to have increased risk of CVI (Galli et al., 2023; Cohen et al., 2007), but this has not been studied systematically. The 15q11.2-q13.1 region is implicated in multiple conditions because it is vulnerable to recombination due to a high density of low copy repeats in this region (DiStefano et al., 2016). Angelman syndrome (AS) and duplication of 15q (Dup15q) syndrome, which are associated with loss or gain of maternally derived genes in the 15q11-13 region, respectively, have overlapping phenotypes including intellectual disability, abnormal speech development, seizures, behavioral disturbances, and features of autism spectrum disorder (ASD) (DiStefano et al., 2016; DiStefano et al., 2020b; Al Ageeli et al., 2014; Bisba et al., 2024). However, visual characteristics, especially features of CVI, are less well described in children with Angelman and Dup15q syndrome (collectively termed 15q disorders).

It can be difficult for clinicians to determine whether functional challenges in children with neurodevelopmental delays (including those with 15q disorders) are related to CVI, unless visual acuity loss is moderate or severe. This is because of limitations of current methods of visual assessment in children with suspected CVI (Musleh et al., 2024; Chang and Borchert, 2021a). Standard pediatric ophthalmologic examination techniques, such as assessing whether a child can fixate and follow objects of various sizes at various distances, can detect decreased visual acuity (Huo et al., 1999; Handa et al., 2018). However, assessment of higher visual processing deficits that can occur in CVI requires specialized testing that is not performed during a routine pediatric ophthalmology examination (Chang and Borchert, 2021a; Chang and Borchert, 2020). Standardized tests of visual perception, such as the Motor-Free Visual Perception Test (MVPT) (Colarusso and Hammill, 1972) and Test of Visual Perceptual Skills (TVPS) (Martin, 2017), frequently administered by neuropsychologists or other professionals with specialized training, generally require a developmental age of at least 3 or 4 years in order for examinees to understand and respond to questions. Parent questionnaires such as the Higher Visual Function Question Inventory (HVFQI) can elicit behaviors suggestive of CVI (Chandna et al., 2021; Macintyre-Beon et al., 2012; Ben Itzhak et al., 2020; Hokken et al., 2025a; Hokken et al., 2025b), but these questionnaires are inherently subjective. Functional vision assessments, such as the CVI Range and Perkins CVI Protocol (Chang et al., 2026; Chang et al., 2022; Zatta and Williems, 2024), can characterize visual challenges in children with CVI, but they are not used for diagnosis. Therefore, there is currently a major clinical gap in diagnosis of CVI in children with developmental delays and neurogenetic syndromes: an objective, validated method that can detect higher visual processing challenges and is applicable to children with a developmental age below 3 years is needed.

In our previous studies, we developed an eye tracking technique suitable for detection and characterization of visual attention to lower- and higher-level visual features in young and developmentally delayed children with CVI (Matsunaga et al., 2024; Matsunaga et al., 2026). Our technique was based on prior literature demonstrating that eye tracking can detect abnormalities of eye movements and visual attention in individuals with CVI, which may aid in distinguishing CVI from other neurodevelopmental disorders (Hokken et al., 2024; Hokken et al., 2023; Manley et al., 2022; Manley et al., 2023; Kooiker et al., 2018). Our lab’s passive eye tracking protocol does not require children to follow any directions other than watching images on a computer monitor; thus, we have been able to successfully collect eye tracking data in developmentally delayed children as young as 12 months of age. In the Vision In SyndrOmic Neurodevelopmental disorders-15q (VISiON-15q) study, we performed comprehensive ophthalmologic and behavioral assessment and eye tracking in children with Angelman and Dup15q syndromes. We asked whether children met clinical criteria for CVI and whether eye tracking could detect additional children with suspected CVI. We also asked whether features of CVI related to vision-specific quality of life, overall development, and adaptive function in children with 15q disorders. The goals of our study were to (1) determine whether eye tracking could improve detection of CVI beyond standard ophthalmologic examination in children with 15q disorders and (2) assess whether CVI was associated with functioning in non-visual domains. The latter goal was included because it could inform the design of future studies to assess the impact of CVI interventions on overall behavior, function, and quality of life in children with Angelman and Dup15q syndromes, as well as other neurogenetic disorders.

## 2 Methods

This study was approved by the local institutional review board (IRB) and adhered to the tenets of the Declaration of Helsinki and the US Health Insurance Portability and Accountability Act of 1996. Informed consent was obtained from the parent or legal guardian of all participants.

### 2.1 Inclusion and exclusion criteria

We recruited 25 children between 12 months to 12 years of age with a genetically confirmed diagnosis of Angelman Syndrome or Dup15q Syndrome from the CHLA multidisciplinary neurogenetics and pediatric neuro-ophthalmology clinics. Exclusion criteria included non-English speaking families (due to psychometrist and questionnaire availability in English only), photosensitive epilepsy (to prevent seizures during eye tracking), any intraocular pathology such as optic atrophy, nystagmus, and any oculomotor disorder such as oculomotor apraxia that would preclude accurate assessment of visual acuity based on visual behavior. We did include children with strabismus, as we have previously published methods to perform eye tracking in children with strabismus (Matsunaga et al., 2024; Matsunaga et al., 2026; Chang and Borchert, 2021b; Chang and Borchert, 2024; Chang and Borchert, 2025).

### 2.2 Ophthalmology examination

Complete pediatric ophthalmologic examination was performed in all participants. Visual acuity was assessed with participants wearing their appropriate refractive correction. Optotype acuity was performed if possible based on age, cognitive status, and visual function. If unable to perform optotype acuity, Teller acuity card testing was attempted. Teller acuity testing was performed at a distance of 55 cm, starting at 0.23 cycles/cm and progressing in a standard two-up, one-down staircase. Each card was presented three times, and the grating was considered “seen” if a saccade or head turn was made toward the side of the grating at least twice. The highest frequency grating that was seen was recorded as the Teller acuity. If the child did not preferentially look toward the lowest frequency card (0.23 cycles/cm) after three attempts, the Teller acuity was recorded as “unable.”

In all participants, visual acuity was also assessed using the 6-level Visual Behavior Scale (VBS, Supplementary Table 1) (Huo et al., 1999; Handa et al., 2018; Chang and Borchert, 2021b). A pediatric neuro-ophthalmologist (MYC) recorded the child’s ability to fixate and follow objects of various sizes at varying distances. In the VBS, a higher score indicates better visual acuity.

For all methods of visual acuity assessment, binocular visual acuity testing was performed, followed by monocular testing if possible.

We also evaluated fixation preference, pupils, ocular motility, and the anterior and posterior segments of the eye, in addition to cycloplegic refraction. Ocular alignment was assessed with alternate cover testing with prisms if possible, but most patients required measurement using the Krimsky test due to challenges with cooperation. Strabismus was diagnosed in patients with any manifest ocular deviation. Amblyopia was diagnosed in patients with asymmetric visual acuity (≥0.2 logMAR interocular acuity difference if quantitative visual acuity testing could be performed), in the setting of an amblyopia risk factor (strabismus or anisometropia; there were no patients with risk factors for deprivation amblyopia). Refractive errors were considered significant if the AAO Preferred Practice Pattern criteria for spectacle correction were met (Hutchinson et al., 2023).

### 2.3 Higher visual function question inventory (HVFQI)

The HVFQI is a structured history-taking questionnaire designed to elicit behaviors suggestive of higher visual processing challenges in individuals with CVI (Chandna et al., 2021). There are two pediatric versions for children 0–3 years of age and 4–18 years of age. We administered the questionnaire to parents via an online web portal. The HVFQI version was selected based on the child’s chronological age.

### 2.4 Developmental, adaptive behavior, and vision-specific quality of life assessments

Cognitive, language and motor development were assessed using the Mullen Scales of Early Learning (MSEL) (Mullen, 1995). All five MSEL scales were assessed: Visual Reception, Gross and Fine Motor, and Expressive and Receptive Language. In order to generate scores for participants outside the normed age range, Developmental Quotients were calculated as follows: (developmental age) / (chronological age) x 100. The Expressive and Receptive Language scales were combined to calculate a Verbal Developmental Quotient. All participants completed the MSEL regardless of chronological age in order to avoid floor effects inherent when administering age-appropriate measures to children with moderate-to-severe intellectual disability (DiStefano et al., 2020a).

Parents were administered the Vineland Adaptive Behavior Scales, 3rd ed. (VABS-III) (Sparrow et al., 2016), a standardized interview measure of adaptive function, and the Children’s Visual Function Questionnaire (CVFQ) (Birch et al., 2007), a validated pediatric vision-specific quality of life (QOL) scale. The VABS-III evaluates adaptive functioning skills across multiple domains, including communication, daily living skills, socialization, and motor function. VABS-III composite and domain scores were reported as standard scores with a mean of 100 and standard deviation (SD) of 15. VABS-III subdomain scores were reported as v-scale scores with a mean of 15 and SD of 3. The CVFQ provides scores in 3 domains (Competence, Personality, and Family Impact), in addition to a total score. CVFQ scores range from 0 to 1, with 1 indicating better vision-specific QOL.

### 2.5 Eye tracking

#### 2.5.1 Eye tracking recordings

We performed eye tracking as previously described (Matsunaga et al., 2024; Matsunaga et al., 2026; Chang and Borchert, 2021b; Chang and Borchert, 2024; Chang and Borchert, 2025). Consistent with our prior studies, a VBS score of at least 4 on the 6-level VBS (Supplementary Table 1) was required to perform eye tracking (in order to view images on the computer monitor). Participants wore their habitual spectacles and were seated 60 cm from a computer monitor with an attached SR Research EyeLink 1,000 (Ottawa, Canada) eye tracker to monitor the direction of eye gaze. We recorded at 500 Hz. Recording was performed binocularly in patients without strabismus; in patients with strabismus, the fixating eye was recorded. The recording session began with three-point calibration, followed by participants viewing still images and videos on the computer monitor for 10 min with no specific task instructions (“free viewing”). The experimental protocol was designed to assess multiple aspects of visual processing in children with CVI and included preferential looking stimuli as well as realistic and cartoon images and videos to assess visual attention. Eye tracking visual acuity was assessed as previously reported (Chang and Borchert, 2021b; Chang and Borchert, 2024), using preferential looking stimuli consisting of a series of high-contrast black-and-white vertical square-wave gratings presented randomly to the right or left side of the screen on a luminance-matched gray background. The grating frequency ranged from 0.25 to 20 cycles per degree (cpd). Each grating was presented for 2 s. The series of gratings was presented three times, with pictures and videos interspersed to maintain attention.

#### 2.5.2 Eye tracking data analysis

##### 2.5.2.1 Labeling fixations and saccades in eye tracking recordings

Recordings were excluded if the calibration error was greater than 2.5 degrees, consistent with prior studies in children with CVI and other neurodevelopmental disorders (Matsunaga et al., 2026; Shic et al., 2022). The SR research eye tracker recorded the X and Y coordinates of each eye at 500 Hz and parsed the data into fixations and saccades. Fixations were defined as events when the eyes moved less than 0.1 degrees over a duration of at least 100 milliseconds. Events were labeled as saccades when eye velocity exceeded 30 degrees/s, acceleration was greater than 8,000 degrees/s^2^, and amplitude was above 0.1 degrees.

##### 2.5.2.2 Visual acuity assessment by eye tracking

Eye tracking visual acuity testing was based on preferential looking to gratings of increasing frequency (Chang and Borchert, 2021b; Chang and Borchert, 2024). Using the SR Research data analysis software, DataViewer (version 4.3, Ottawa, Canada), we labeled the gratings as “interest areas.” For each participant, during each trial with gratings presented, the percentage of fixations that were located within the grating interest area was calculated. A grating was considered “seen” when this metric exceeded 50%. The grating acuity threshold was defined as the highest frequency grating that was preferentially viewed at least twice (out of three trials). Eye tracking visual acuity was considered abnormal if below the lower end of the 95% normative prediction limits of grating acuity by age (Mayer et al., 1995; Drover et al., 2009), or below 20 cpd (the maximum grating acuity in our eye tracking protocol) if the lower limit of the normative range was greater than 20 cpd.

##### 2.5.2.3 Saliency analysis of Eye Tracking for CVI (SET-CVI)

We performed saliency analysis, a computer vision technique that assesses which aspects of an image attract a viewer’s attention, using our novel SET-CVI technique (Matsunaga et al., 2024; Matsunaga et al., 2026). SET-CVI quantifies visual attention to 15 lower- and higher-level visual characteristics and generates eye tracking signatures that can be used to identify an individual’s relative strengths and weaknesses in visual attention. Normative ranges in typically developing (TD) pediatric controls have previously been published (Matsunaga et al., 2026). The SET-CVI signatures in children with CVI demonstrate decreased fixations to visual characteristics requiring higher-level processing and increased fixations to certain lower-level visual characteristics (Matsunaga et al., 2026).

The steps involved in performing SET-CVI have been previously published (Matsunaga et al., 2024; Matsunaga et al., 2026; Avramidis et al., 2024). We first excluded participants with eye tracking visual acuity worse than 3 cpd, which is the minimum visual acuity required to resolve >99% of the spatial frequencies represented in the images within our experimental protocol. Next, we created saliency maps for each image in the eye tracking protocol using a combination of pixel-based and generative artificial intelligence techniques to highlight 15 lower- and higher-level visual characteristics, such as certain colors, orientation, luminance, human bodies, and human faces (for full list, see Supplementary Figure 1). For each image in the eye tracking protocol, each participant’s eye tracking fixation map was compared to the saliency maps in order to calculate fixation saliency values (Supplementary Figure 2). Fixation saliency values ranged from 0 to 255 and corresponded to the pixel intensity on the saliency map in the location of the fixation. Higher fixation saliency values indicated more fixations (and greater visual attention) to the visual characteristic highlighted on the saliency map. For each participant, average fixation saliency values to each of the 15 lower- and higher-level visual characteristics were compared to the previously published normative range (Matsunaga et al., 2026) and labeled as “normal, “above normal,” or “below normal.” The SET-CVI eye tracking signature consisted of the pattern of normal and abnormal fixation saliency values for each participant.

### 2.6 CVI diagnostic categories

#### 2.6.1 CVI diagnosis

CVI was diagnosed clinically based on the NIH CVI working definition (Chang et al., 2024). Children were required to have objective evidence of visual dysfunction that could not be explained by any ocular pathology. We diagnosed CVI with reduced visual acuity based on binocular VBS score < 5 (Supplementary Table 1) or binocular Teller or eye tracking acuity below the lower limit of the 95% normative prediction interval for age (Mayer et al., 1995; Drover et al., 2009). We attempted to perform standardized tests of visual perception in all participants, but only one child was able to complete this assessment. Therefore, we used the results of the HVFQI and eye tracking saliency analysis (see below) to identify 15q children who had suspected CVI with higher-level visual processing deficits and good visual acuity. We considered these children to be CVI “suspects” since eye tracking saliency analysis has not been validated for diagnosing CVI in this population, and HVFQI scores are subjective.

#### 2.6.2 CVI suspects

##### 2.6.2.1 CVI suspects based on HVFQI scores

The validation study of the HVFQI demonstrated that this questionnaire could discriminate between children with CVI and typically developing control children (Chandna et al., 2021). However, there is no published cut-off for the HVFQI to differentiate between children with neurogenetic disorders who have CVI from developmentally delayed children without CVI. The HVFQI version for 4–18 year olds is derived from the CVI Inventory, which includes Five Questions that have been used for CVI screening in children with neurodevelopmental disorders (Macintyre-Beon et al., 2012; Gorrie et al., 2019). Therefore, in children 4 years of age and older, we extracted the responses from these Five Questions to determine if they met criteria for suspected CVI (3 or more of the Five Questions endorsed). There are no published thresholds to detect CVI using the HVFQI for 0–3 year olds. Therefore, for children 3 years of age and younger, we considered them to be CVI suspects if their score was above the median HVFQI 0–3 score in our study.

##### 2.6.2.2 CVI suspects based on eye tracking saliency analysis

In order to identify 15q children with abnormal visual attention suggestive of CVI with higher-order visual deficits using eye tracking, we analyzed the fixation saliency values calculated using SET-CVI (Supplementary Figure 2). We first excluded 15q children diagnosed with CVI clinically, since eye tracking would not be needed to identify CVI in these patients. We also excluded children with poor calibration and eye tracking acuity worse than 3 cpd. Since there is no established threshold of abnormal fixation saliency values in children with 15q disorders, we performed unsupervised, data-driven machine learning analysis to separate 15q participants’ eye tracking recordings into groups. Specifically, we performed hierarchical clustering using the web-based tool, clustVis^1^ (Metsalu and Vilo, 2015). Clustering was performed using Euclidean distance and Ward linkage, and recordings were divided into two groups based on the highest level of partitioning (first split in the dendrogram). We then calculated the number of abnormal SET-CVI features in each group. The children in the group with the higher number of abnormal SET-CVI characteristics were considered to have “high suspicion” of CVI (15q-CVI-HS), and the participants in the group with fewer abnormal characteristics on SET-CVI were considered “low suspicion” of CVI (15q-CVI-LS).

We then compared the eye tracking fixation saliency values in the 15q-CVI-HS and 15q-CVI-LS group to previously recruited children diagnosed with CVI (none of whom had 15q disorders) and typically developing (TD) control children (Matsunaga et al., 2026). In our previous studies, we performed the same eye tracking protocol in 43 children with CVI (mean age 4.3 ± 2.9 years) and 40 TD controls (mean age 5.6 ± 3.4 years) between the ages of 12 months and 12 years (Matsunaga et al., 2026). Comparisons between groups on fixation saliency values to 15 lower- and higher-level visual characteristics were performed using multivariate regression to adjust for age and sex.

#### 2.6.3 CVI diagnosis or high suspicion category (15q-CVI-DHS)

As described above, we identified 3 groups of CVI participants: children diagnosed with CVI, children who were CVI suspects based on eye tracking and/or HVFQI results, and children with low suspicion for CVI (15q-CVI-LS). We reviewed the results of developmental, adaptive behavior, and vision-specific quality of life measurements and found that scores in the CVI diagnosis and CVI suspect groups were not significantly different (*p* ≥ 0.13). Therefore, for the purposes of group comparisons below, we combined the CVI diagnosis and CVI suspect groups into a single category, labeled 15q-CVI-DHS. This was a pragmatic decision due to limited sample size, which also may have precluded detecting any significant differences between the children with diagnosed and suspected CVI.

### 2.7 Comparison of 15q-CVI-LS and 15q-CVI-DHS groups

#### 2.7.1 Demographics and clinical characteristics

We compared the 15q-CVI-LS and 15q-CVI-DHS groups on demographics and clinical characteristics. In addition to ophthalmologic diagnoses, we also assessed rates of neurologic conditions including seizures, hypoxic–ischemic encephalopathy, and cerebral palsy. Groups were compared using Mann Whitney tests for continuous variables and Fisher’s exact test for categorical variables.

#### 2.7.2 Visual reception

We compared scores on the Visual Reception scale of the MSEL in children in the 15q-CVI-LS and 15q-CVI-DHS groups using the Mann–Whitney test.

#### 2.7.3 Development, adaptive behavior, and vision-specific quality of life

We compared 15q-CVI-LS and 15q-CVI-DHS groups on non-visual MSEL scales and VABS-III composite, domain, and subdomain scores using Mann–Whitney tests. For CVFQ scores, we compared groups using multivariate regression to adjust for chronological age and sex since standard scores were not available. We also included strabismus and amblyopia in the multivariate regression model because these ophthalmologic conditions have been associated with decreased vision-specific quality of life (Birch et al., 2007; Leske et al., 2021; Buffenn, 2021).

Statistical analyses were performed using Prism 10.3.1 (GraphPad Software, LLC, San Diego, CA). *p*-values less than 0.05 were considered significant. Since CVI has not previously been systematically studied in children with Angelman and Dup15q syndromes, all analyses were considered exploratory and no correction for multiple comparisons was applied.

## 3 Results

### 3.1 Participants

We enrolled 25 children with 15q disorders whose demographics and clinical characteristics are shown in Table 1. Based on clinical assessment and eye tracking, we classified 15 participants as 15q-CVI-DHS (detailed description below). Nine children were considered to have low suspicion of CVI (15q-CVI-LS). One child had a normal VBS score and could not perform Teller acuity or accurate eye tracking calibration; additionally, the parent did not complete the HVFQI. The CVI assessment was considered inconclusive for this participant, who was excluded from analyses comparing 15q-CVI-DHS and 15q-CVI-LS groups. The mean age was 5.7 ± 3.2 years, not significantly different between the 15q-CVI-LS and 15q-CVI-DHS groups. There was no difference between groups in sex, race, ethnicity, and comorbid neurologic conditions including seizures, hypoxic–ischemic encephalopathy, and cerebral palsy. Overall, 40% of 15q children had strabismus, 8% had amblyopia, and 16% had significant refractive errors, with no significant differences between groups in rates of ophthalmologic conditions. None of the participants had any intraocular pathology, such as retinopathy of prematurity (ROP) or optic atrophy. Additionally, none of the participants were taking vigabatrin.

**Table 1:** Demographics and clinical characteristics of 25 children with 15q disorders, divided into children with CVI diagnosis or high suspicion (15q-CVI-DHS) and CVI low suspicion (15q-CVI-LS).

Characteristic	All 15q *N* = 25	15q-CVI-DHS *N* = 15	15q-CVI-LS *N* = 9	CVI-indeterminate *N* = 1	*p*-value 15q-CVI-DHS vs. 15q-CVI-LS
Demographics					
Age (years)	5.7 ± 3.2	6.1 ± 3.5	5.6 ± 2.7	8.8	0.99
Sex (male/female)	9 (36%) / 16 (64%)	5 (33%) / 10 (67%)	3 (33%) / 6 (67%)	0/1 (100%)	>0.99
Race/ethnicity					0.71
Asian	2 (8%)	2 (13%)	0	0	
Black	0	0	0	0	
Hispanic	5 (20%)	2 (13%)	3 (33%)	0	
Non-Hispanic white	8 (32%)	5 (33%)	3 (33%)	1 (100%)	
Mixed/other/decline	10 (40%)	6 (40%)	3 (33%)	0	
Genetic and neurologic conditions					
Angelman syndrome	5 (20%)	3 (20%)	2 (22%)	0	0.40
Deletion	3 (60%)	1 (7%)	2 (22%)	0	
Unknown	2 (40%)	2 (13%)	0	0	
Dup15q syndrome	20 (80%)	12 (80%)	7 (78%)	1 (100%)	0.04*
Interstitial	3 (15%)	0	3 (33%)	0	
Isodicentric (idic)	17 (85%)	12 (80%)	4 (44%)	1 (100%)	
Seizures	15 (60%)	8 (53%)	6 (67%)	1 (100%)	0.67
HIE	2 (8%)	1 (7%)	1 (11%)	0	>0.99
Cerebral palsy	1 (4%)	0	1 (11%)	0	0.38
Ophthalmic conditions					
Strabismus	10 (40%)	8 (53%)	2 (22%)	0	0.21
Amblyopia	2 (8%)	2 (13%)	0	0	0.51
Significant refractive error	4 (16%)	4 (27%)	0	0	0.26
Visual acuity					
Visual behavior scale (VBS) score	5.5 ± 1.0	5.2 ± 1.3	5.9 ± 0.33	6	0.14
Teller or optotype acuity					
Unable to perform	14 (56%)	6 (40%)	7 (78%)	1 (100%)	0.10
logMAR acuity	1.0 ± 0.66	1.11 ± 0.66	0.43 ± 0.33	N/A	0.18

One child was considered to have indeterminate CVI status due to incomplete assessments.

*p < 0.05.

HIE, hypoxic–ischemic encephalopathy.

Of the 25 total participants, 5 (20%) children had AS and 20 (80%) children had Dup15q Syndrome. Three of the AS children had deletions, and the genetic subtypes of the two remaining AS children were unknown because they were diagnosed outside of our institution and detailed genetic test results were not available. Of the 20 children with Dup15q Syndrome, 3 (15%) had interstitial duplications and 17 (85%) had the isodicentric (idic) subtype. All Dup15q children with interstitial duplications were categorized as 15q-CVI-LS, whereas only 4 of 17 (24%) children with the idic subtype were categorized as 15q-CVI-LS (*p* = 0.04, Table 1). Additional demographic and clinical characteristics of children with AS and Dup15q Syndrome are provided in Table 2. There was no significant difference between AS and Dup15q groups on demographics, rates of neurologic and ophthalmic conditions, and best-corrected visual acuity, except that children with AS were more likely to have a significant refractive error corrected by spectacles (60% vs. 5%, *p* = 0.016). There was no significant difference between children with AS and Dup15q syndrome in percentages of participants classified as 15q-CVI-DHS, 15q-CVI-LS, or CVI-indeterminate (*p* > 0.99). Therefore, we combined children with AS and Dup15q syndrome in the following analyses; however, we separately indicated children with the two disorders in the figures.

**Table 2:** Demographics and clinical characteristics of 5 children with Angelman syndrome and 20 children with Duplication of 15q (Dup15q) syndrome, including categorization by CVI diagnosis or suspicion.

Characteristic	Angelman syndrome (*n* = 5)	Dup15q syndrome (*n* = 20)	*p*-value
Demographics			
Age (years)	5.4 ± 3.7	5.8 ± 3.2	0.73
Sex (male/female)	1 (20%) / 4 (80%)	8 (40%) / 12 (60%)	0.62
Race/ethnicity			0.79
Asian	1 (20%)	1 (5%)	
Black	0	0	
Hispanic	1 (20%)	4 (20%)	
Non-Hispanic white	1 (20%)	7 (35%)	
Mixed/other/decline	2 (40%)	8 (40%)	
Neurologic conditions			
Seizures	4 (80%)	11 (55%)	0.61
HIE	1 (20%)	1 (5%)	0.37
Cerebral palsy	0	1 (5%)	>0.99
Ophthalmic conditions			
Strabismus	3 (60%)	7 (35%)	0.36
Amblyopia	1 (20%)	1 (5%)	0.37
Significant refractive error	3 (60%)	1 (5%)	0.016*
Visual acuity			
Visual behavior scale (VBS) score	5.6 ± 0.55	5.5 ± 1.1	>0.99
Teller or optotype acuity			
Unable to perform	3 (60%)	11 (55%)	>0.99
logMAR acuity	1.2 ± 1.3	0.94 ± 0.56	>0.99
CVI diagnosis or suspicion group			
15q-CVI-DHS	3 (60%)	12 (60%)	>0.99
CVI clinical diagnosis	1 (33%)	7 (64%)	
Decreased VA on eye tracking	1 (33%)	1 (9%)	
Abnormal visual attention on SET-CVI	1 (33%)	3 (27%)	
Abnormal functional vision questionnaire (HVFQI)	0	1 (5%)	
15q-CVI-LS	2 (40%)	7 (35%)	
CVI-indeterminate	0	1 (5%)	

HIE, hypoxic–ischemic encephalopathy; 15q-CVI-DHS, CVI diagnosis or high suspicion; 15q-CVI-LS, CVI low suspicion; VA, visual acuity, SET-CVI, Saliency analysis of Eye Tracking in CVI; HVFQI, Higher Visual Function Question Inventory; *p < 0.05.

### 3.2 Classification into 15q-CVI-DHS and 15q-CVI-LS groups

#### 3.2.1 15q-CVI-DHS group designation based on decreased visual acuity on clinical exam

Of the 15 participants in the 15q-CVI-DHS group, eight (53%) were diagnosed with CVI based on reduced binocular visual acuity for age on clinical examination. Visual acuity was decreased on VBS testing in two participants (VBS scores 2 and 3). In six children, VBS score was normal, but Teller acuity was below the lower bound of the 95% normative prediction limits for age. This discrepancy is due to limited granularity of the VBS scale, which leads to difficulties in detecting mild to moderate levels of decreased visual acuity.

A reliable Teller acuity score was not achieved in 14 (56%) participants due to challenges with cooperation or attention to the task. Only one participant (4%) was able to perform optotype acuity testing.

#### 3.2.2 15q-CVI-DHS group designation based on decreased eye tracking visual acuity

Two children with normal VBS scores could not cooperate with Teller acuity testing, but were able to perform accurate eye tracking calibration and were found to have reduced binocular visual acuity for age during eye tracking. These children were categorized as 15q-CVI-DHS.

#### 3.2.3 15q-CVI-DHS group designation based on eye tracking saliency analysis

Sixteen 15q participants (3 with CVI diagnosis) had good calibration on eye tracking and sufficient visual acuity for saliency analysis (SET-CVI). As described above, for each participant, we calculated fixation saliency values (FSVs) to 15 lower- and higher-level visual characteristics (Supplementary Figure 2). The 13 participants who underwent SET-CVI and did not have a CVI diagnosis underwent clustering analysis. Hierarchical clustering results based on eye tracking FSVs are shown in Figure 1. We identified two groups of 15q participants based on eye tracking saliency analysis. Group 2 (*n* = 4) was characterized by higher FSVs to lower-level visual features such as certain colors and luminance, and lower FSVs to features requiring higher-level visual processing to interpret, such as still depictions of motion and two-dimensional depictions of depth. This profile is consistent with previously described saliency patterns in children with CVI (Matsunaga et al., 2026; Walter et al., 2024).

**Figure 1:**
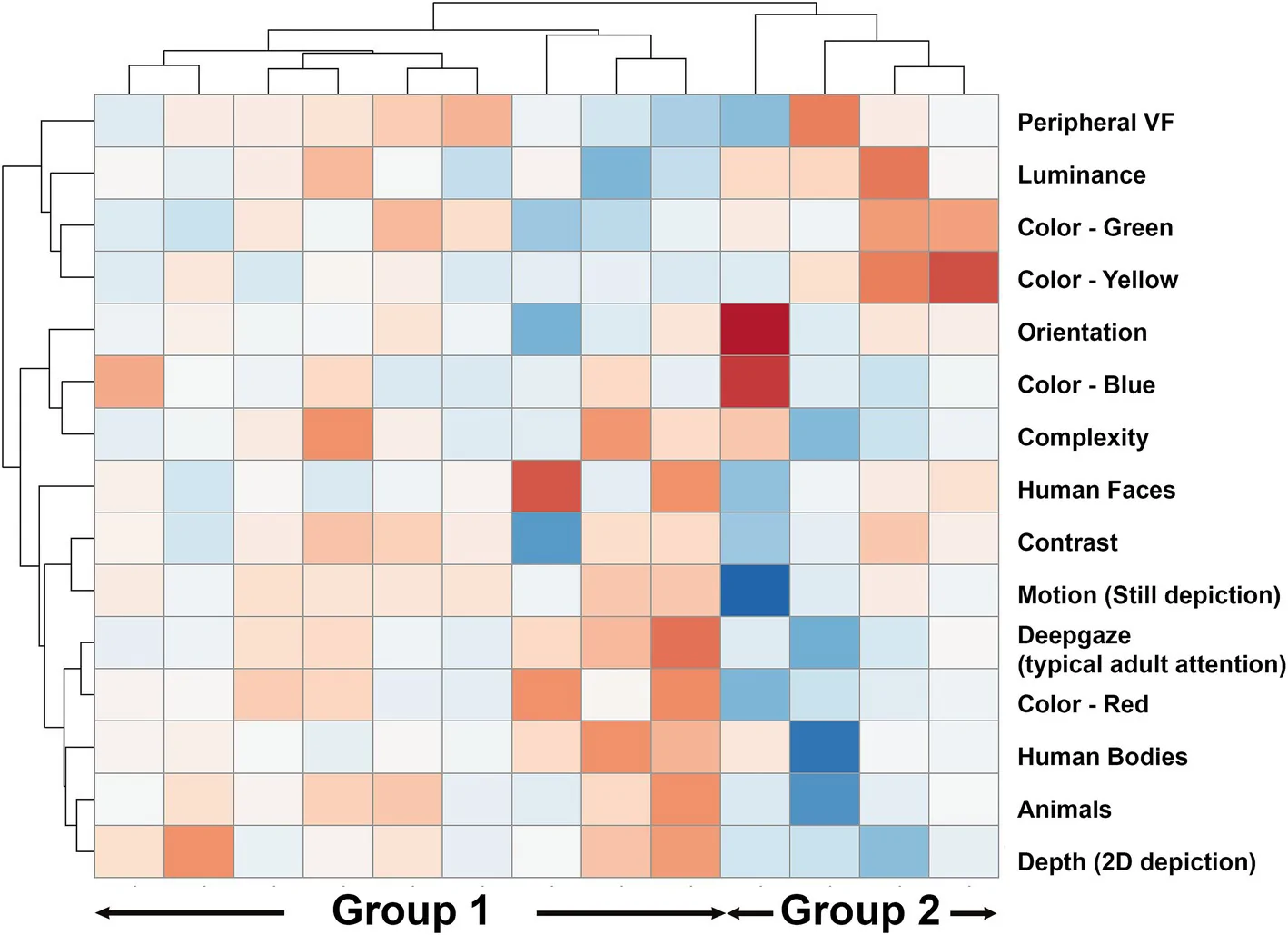
Hierarchical clustering dendrogram to identify two groups of 15q children based on eye tracking fixation saliency values. Each column represents one participant, and each row represents one of 15 lower- and higher-level visual characteristics. Warmer colors indicate higher fixation saliency values to the visual characteristics, and cooler colors indicate lower fixation saliency values to the visual characteristics.

We generated SET-CVI eye tracking signatures for children in groups 1 and 2, as well as the three children with CVI diagnosis who had sufficient visual acuity for saliency analysis (Figure 2). The mean number of abnormal features on SET-CVI was 2.9 ± 1.8 in group 1, 8.0 ± 2.4 in group 2, and 8.7 ± 4.0 in the 15q CVI diagnosis group (*p* = 0.0056, group 1 vs. 2; *p* = 0.02, group 1 vs. 15q CVI diagnosis group). Since there were fewer abnormal SET-CVI features in group 1, this group was considered “low suspicion” of CVI (15q-CVI-LS). Group 2 was considered “high suspicion” of CVI (15q-CVI-HS).

**Figure 2:**
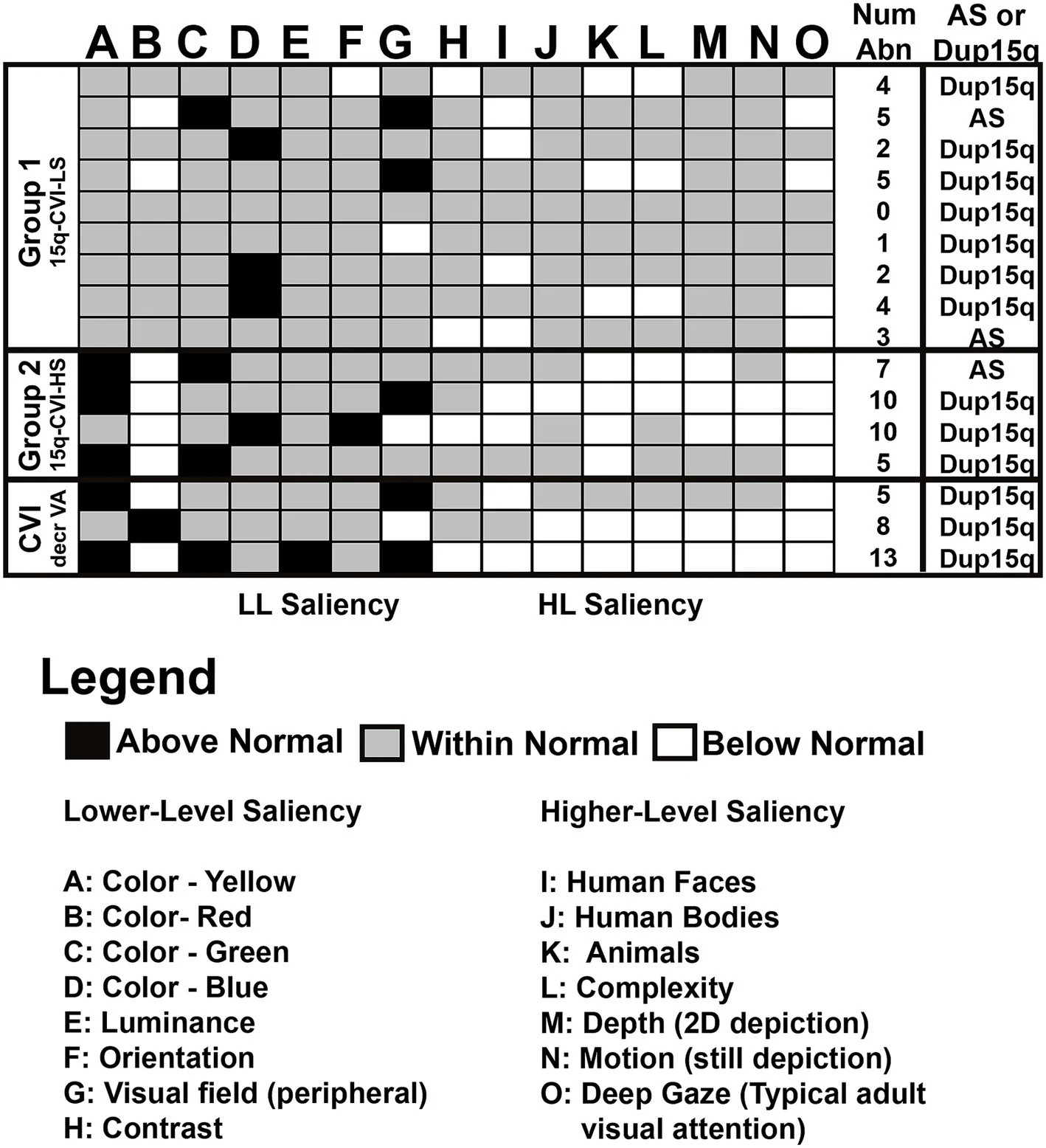
Saliency analysis of Eye Tracking for CVI (SET-CVI) signatures in children in eye tracking groups 1 and 2. Based on higher number of abnormal (Num Abn) SET-CVI features in group 2, this group was considered “high suspicion” for CVI (15q-CVI-HS). Group 1 was labeled “low suspicion” of CVI (15q-CVI-LS) due to fewer abnormal SET-CVI characteristics. Additionally, SET-CVI signatures for 3 children with CVI diagnosis who had visual acuity that was decreased for age (decr VA) but sufficient to perform saliency analysis are shown. Genetic diagnosis of Angelman Syndrome (AS) or Duplication of 15q Syndrome (Dup15q) is provided for each participant.

To further justify the designation of group 2 as “high suspicion” of CVI, we compared the 15q-CVI-LS and 15q-CVI-HS groups to previously recruited children with CVI diagnosis (and without a 15q disorder) and TD controls. Fixation saliency values to 15 lower- and higher-level visual characteristics in the 4 groups are shown in Figure 3. In general, children in the 15q-CVI-HS group were more similar to the CVI group than TD controls, and the opposite was true for the 15q-CVI-LS group. However, the 15q-CVI-LS group did have lower FSVs than TD controls to several visual characteristics requiring higher-level visual processing.

**Figure 3:**
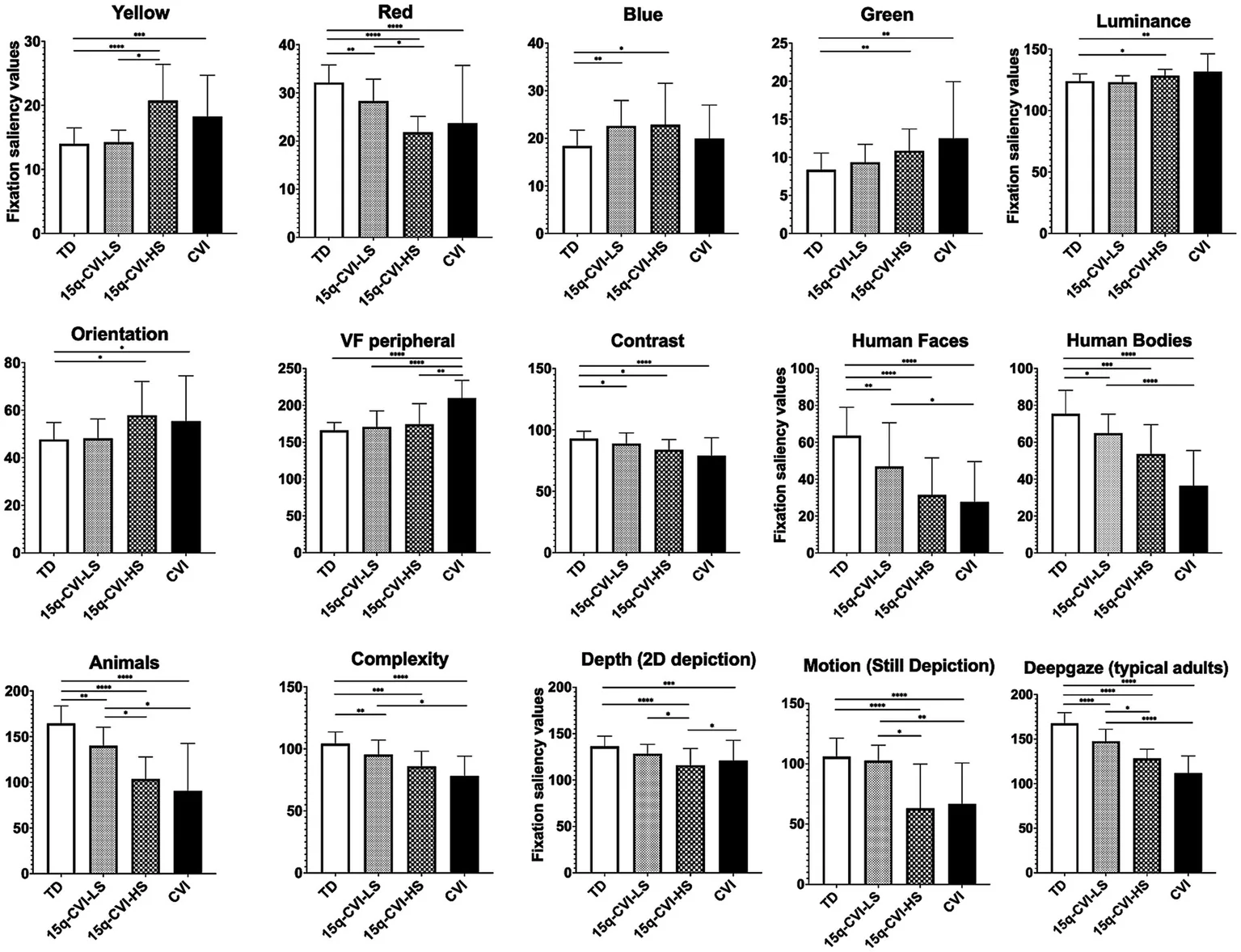
Comparison of 15q-CVI-LS and 15q-CVI-HS eye tracking groups to previously reported typically developing (TD) pediatric controls and children with CVI diagnosis. Fixation Saliency Values indicate visual attention to 15 lower- and higher-level visual characteristics and were calculated during Saliency analysis of Eye Tracking in CVI (SET-CVI) analysis. *p<0.05, **p<0.01, ***p<0.001, ****p<0.0001.

#### 3.2.4 15q-CVI-DHS group designation based on HVFQI scores

One child (7%) was classified as 15q-CVI-DHS based on abnormal HVFQI responses only. This child had a normal VBS score and was unable to perform reliable Teller acuity testing or eye tracking calibration. However, their parent endorsed four of five HVFQI questions used for CVI screening, so the participant was considered a CVI suspect.

#### 3.2.5 Final classification based on CVI diagnosis or suspicion

In total, 15 children were categorized in the 15q-CVI-DHS group. As noted previously, 1 participant had indeterminate CVI status due to incomplete assessments. The remaining 9 participants were classified as CVI-low suspicion (15q-CVI-LS).

### 3.3 Visual reception in 15q-CVI-LS and 15q-CVI-DHS groups

Because of their cognitive status, the majority of our participants were unable to perform standardized tests of visual perception. Therefore, we used the MSEL Visual Reception Scale to characterize visual cognitive processing skills in the 15q-CVI-LS and 15q-CVI-DHS groups. Two participants (8%), one in each CVI group, were unable to complete the MSEL due to challenges with cooperation. In the remaining participants, we found that MSEL Visual Reception Developmental Quotient scores were significantly lower in the 15q-CVI-DHS group than the 15q-CVI-LS group (17.6 ± 12.4 vs. 43.9 ± 25.7, *p* = 0.024, Figure 4, upper row).

**Figure 4:**
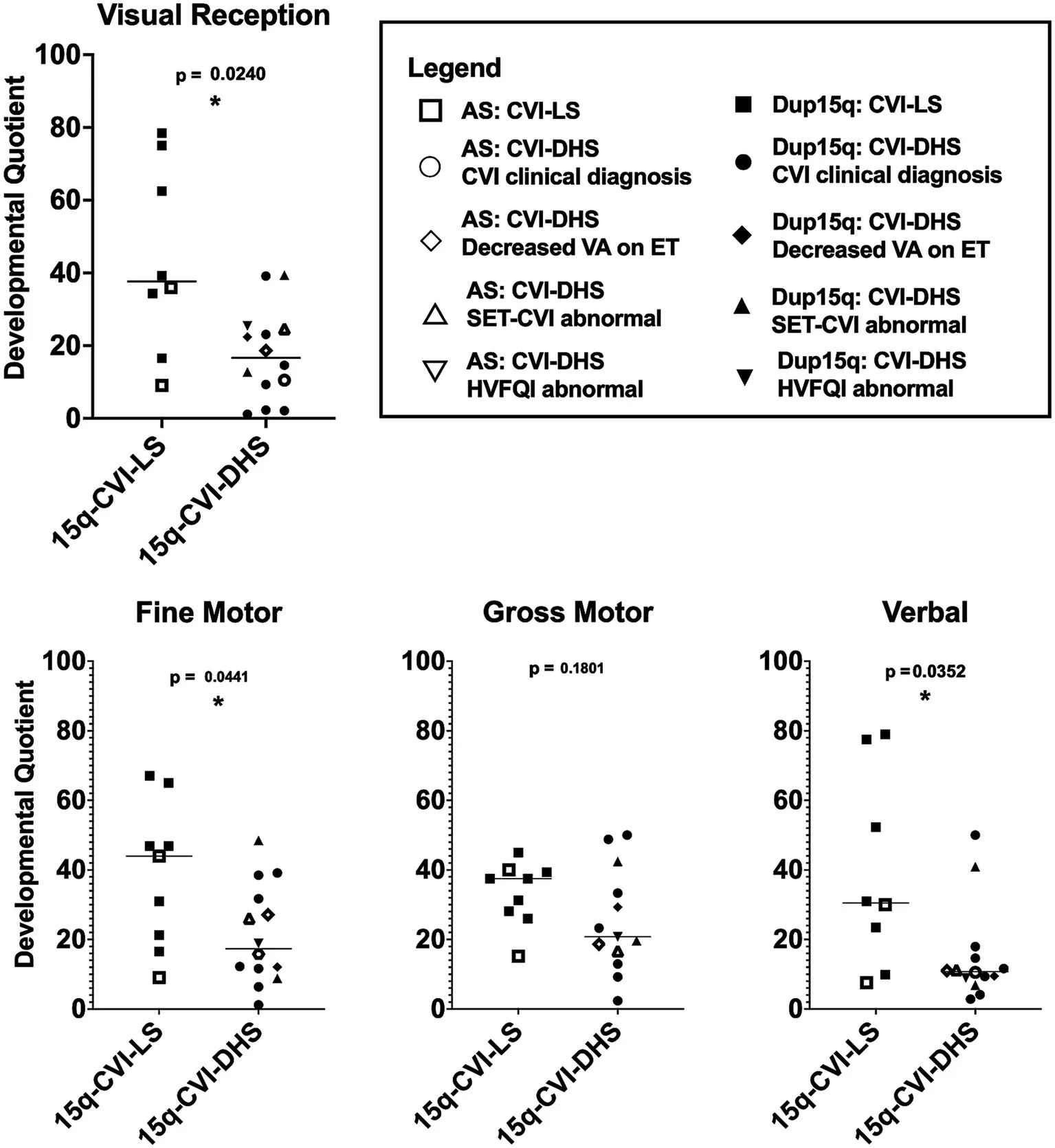
Developmental quotient scores on the Mullen Scales of Early Learning (MSEL) Visual Reception, Fine Motor, Gross Motor, and Verbal scales in 15q children with CVI low suspicion (15q-CVI-LS) and CVI diagnosis or high suspicion (15q-CVI-DHS) groups. *p<0.05.

### 3.4 Development in non-visual domains in 15q-CVI-LS and 15q-CVI-DHS groups

We compared scores on non-visual MSEL scales in the two groups to assess development in other domains. We found that scores were lower in the 15q-CVI-DHS group (Figure 4, lower row). The difference was borderline in the fine motor and verbal (both *p* = 0.04) domains, and non-significant in the gross motor domain.

### 3.5 Adaptive behavior in 15q-CVI-LS and 15q-CVI-DHS groups

The parent or legal guardian of all participants completed the non-motor sections of the VABS-III. The motor skills section of the VABS-III was completed in 22 of 25 (88%) participants. We compared VABS-III composite and domain scores between children in the 15q-CVI-LS and 15q-CVI-DHS groups (Figure 5), and there were no significant differences. However, there were significantly lower v-scale scores in the 15q-CVI-DHS group on the Receptive Communication (2.00 ± 2.36 vs. 6.00 ± 3.71, *p* = 0.0044), Social Interaction (3.33 ± 2.87 vs. 6.11 ± 2.85, *p* = 0.036), and Gross Motor (2.46 ± 2.42 vs. 5.88 ± 3.64, *p* = 0.035) subdomains.

**Figure 5:**
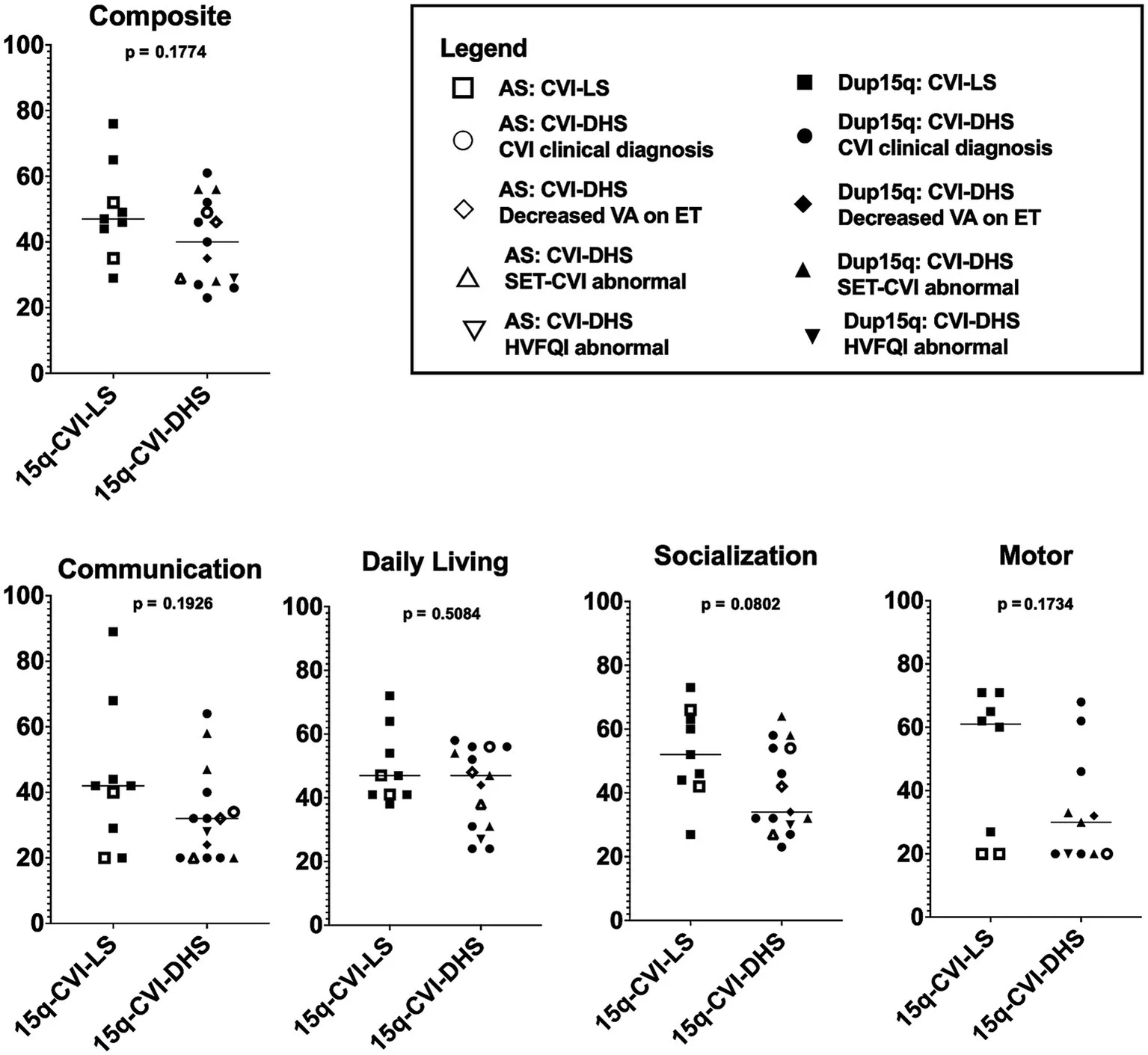
Standard scores on the Vineland Adaptive Behavior Scales, 3rd edition (VABS-III) adaptive behavior composite and four domains in 15q children categorized into CVI low suspicion (15q-CVI-LS) and CVI diagnosis or high suspicion (15q-CVI-DHS) groups.

### 3.6 Vision-specific quality of life in 15q-CVI-LS and 15q-CVI-DHS groups

The CVFQ was completed by the parent or legal guardian of 23 of 25 (92%) participants. CVFQ total and domain scores were compared between groups (Figure 6). After adjusting for age, sex, amblyopia, and strabismus, children in the 15q-CVI-DHS group had significantly worse vision-specific quality of life overall (0.60 ± 0.18 vs. 0.82 ± 0.10, *p* = 0.022) as well as on the Competence (0.57 ± 0.27 vs. 0.93 ± 0.05, *p* = 0.0045) and Personality (0.64 ± 0.15 vs. 0.78 ± 0.10, *p* = 0.013) subscales. There was a trend toward worse Family Impact scores in the 15q-CVI-DHS group (0.64 ± 0.20 vs. 0.82 ± 0.14), which did not reach significance after adjusting for ophthalmic comorbidities (*p* = 0.11).

**Figure 6:**
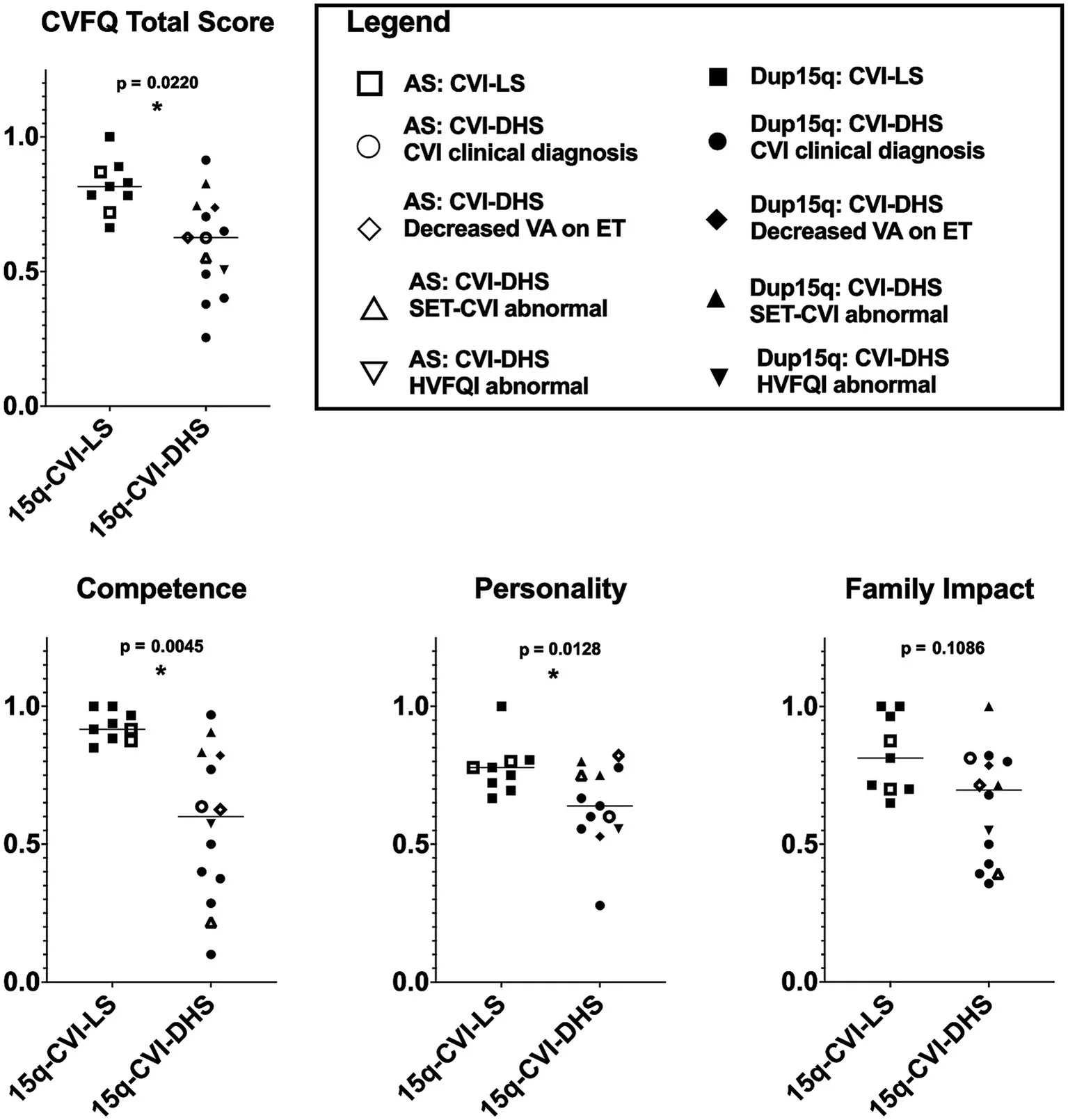
Scores on the Children’s Visual Function Questionnaire (CVFQ) vision-specific quality of life scale in 15q children categorized into CVI low suspicion (15q-CVI-LS) and CVI diagnosis or high suspicion (15q-CVI-DHS) groups. Total and subscale scores are shown. *p<0.05.

## 4 Discussion

Through prospective assessments, including ophthalmologic examinations with Teller acuity testing, functional vision questionnaires, and eye tracking, 15 of 25 (60%) children with AS and Dup15q syndromes were diagnosed with CVI or CVI suspect. CVI was diagnosed or suspected based on clinical examination or Teller acuity testing in 8 (53%) children, eye tracking in 6 (40%) children and HVFQI scores in 1 (7%) child. Thus, nearly half of the patients in our cohort with diagnosed or suspected CVI were identified based on eye tracking alone, indicating that eye tracking can improve the yield of CVI detection in this patient population. Furthermore, CVI features were associated with worse vision-specific quality of life and decreased scores on the MSEL visual reception, verbal, and fine motor scales, suggesting that visual challenges in 15q children with CVI may relate to development in non-visual domains.

### 4.1 Comparison to prior studies of CVI in children with neurogenetic syndromes

Although a 60% rate of CVI may seem high, since CVI has not been historically considered a feature of 15q disorders, this is likely because of the challenges with detecting CVI in children with genetic disorders associated with developmental delays and cognitive impairment. Indeed, prior studies of children with other neurogenetic conditions such as chromosome 8p disorders and CDKL5 deficiency disorder have reported rates of CVI ranging from 54 to 75% (Fasaludeen et al., 2026; Abbott et al., 2025). CVI has not previously been systematically studied in children with AS and Dup15q syndromes. However, when reporting the neurovisual profile of 37 individuals with AS, Galli et al. found decreased visual acuity in 86%, reduced contrast sensitivity in 35%, and oculomotor dysfunctions in 84% of participants (Galli et al., 2023). The authors hypothesized that these visual deficits may be related to CVI; however, a definite diagnosis of CVI was not made. In Dup15q syndrome, there are even fewer data on the visual profile of affected children. In 2006, Cohen et al. reported a case of a 16 year old girl with interstitial Dup15q who had a deficit in visual–spatial skills, which was out of proportion to cognitive testing in other domains (Cohen et al., 2007). At the present time, this child would most likely qualify for a diagnosis of CVI based on the recent NIH CVI working definition (Chang et al., 2024).

### 4.2 Role of genetic subtypes

Detection of CVI in children with interstitial Dup15q is less challenging than in children with the Dup15q idic subtype and Angelman syndrome, since children with an interstitial duplication generally have less cognitive impairment and are more likely to be able to participate with standardized tests of visual perception, as in the case report cited above (Cohen et al., 2007). In our cohort, we found that Dup15q children with the interstitial subtype were significantly less likely to be classified as 15q-CVI-DHS than children with the idic subtype. It is relevant that the more severely cognitively impaired subtype is more likely to display features of CVI, since these patients are less likely to be able to perform standardized tests of visual perception that require children to understand and respond to questions. Thus, CVI assessments that do not require active participation, such as eye tracking, are critical in this population.

Genetic subtypes of AS include deletions, uniparental disomy, pathogenic variants in *UBE3A*, and abnormal imprinting (Duis et al., 2022). Deletions are the most common subtype and are also generally considered the most severe. In our cohort, 3 of 5 AS participants had deletions; the genetic subtype of the remaining 2 was unknown. Thus, we were unable to determine whether AS genetic subtype was associated with differential risk of CVI.

### 4.3 CVI diagnosis by clinical examination with teller acuity testing

Of the 15 children in the 15q-CVI-DHS group, CVI with decreased visual acuity was detected by visual behavior (using the VBS) in only 2 of 15 (13%) cases. VBS scores are widely used clinically since they are fast and efficient, but the lack of sensitivity demonstrated here reflects the insufficient granularity of this test to detect CVI unless visual acuity is moderately or severely reduced. Teller acuity was able to detect an additional 6 (40%) children with reduced visual acuity. However, reliable Teller acuity testing was only possible in approximately half of our participants. The literature is mixed regarding the feasibility of Teller acuity testing in the 15q population. Dickinson et al. and Van Splunder et al. reported that quantitative visual acuity testing was not possible in any individuals with AS in their studies (Dickinson et al., 1990; Van Splunder et al., 2003). However, Galli et al. were successful in measuring quantitative visual acuity with optotypes or Teller acuity in 36 of 37 (97%) individuals with AS (Galli et al., 2023). The reasons for the wide discrepancy are unclear and may relate to differences in age or cognitive ability. In our cohort, the rate of reliable Teller acuity testing was 44%, which is in the middle of these prior reports.

### 4.4 Eye tracking to detect CVI and CVI suspects

Six children in the 15q-CVI-DHS group were classified based on eye tracking. Two had decreased visual acuity on eye tracking that was not detected on the VBS or Teller cards (due to inability to perform reliable Teller acuity testing). Four children were classified as 15q-CVI-DHS based on abnormal visual attention on SET-CVI. Eye tracking to assess visual attention has been performed on a limited basis in children with AS and has not been reported in children with Dup15q syndrome. Hong et al. conducted an eye tracking study in individuals with AS who watched videos that displayed geometric patterns on one side of the screen and social scenes on the other side (Hong et al., 2017). They found that, compared to typically developing controls, individuals with AS demonstrated less preference to social stimuli. In our study, on SET-CVI, 2 of 4 (50%) children in the 15q-CVI-DHS group who were classified based on eye tracking saliency analysis had abnormally low fixations to human faces. Hagenaar et al. performed eye tracking in children with AS, using visual stimuli with varying degrees of saliency presented to the four corners of the screen (Hagenaar et al., 2024; Kooiker et al., 2016). In an exploratory analysis, the authors reported that AS children had slower reaction times compared to typically developing controls when viewing high-saliency stimuli (such as cartoons), and there was less of a difference with low-saliency stimuli (such as colors), indicating less difficulty with stimuli representing lower-level saliency. Consistent with this finding, SET-CVI demonstrated that children in our study who were classified as 15q-CVI-DHS had fewer fixations to higher-level saliency features and more fixations to lower-level saliency features, including certain colors.

The primary goal of the eye tracking study by Hagenaar et al. was to determine whether eye tracking and other measures were feasible as outcome measures in AS (Hagenaar et al., 2024). The authors reported that eye tracking using low- and high-level saliency stimuli to assess visual orienting functions was successfully completed in 89% of participants. For comparison, Hong et al. reported that eye tracking to assess social preference could be performed in only 47% of individuals with AS (Hong et al., 2017). In our study, 18 of 25 (72%) participants were able to complete eye tracking with good calibration, although only 16 (64%) had visual acuity sufficient to perform saliency analysis. Hagenaar et al. achieved their high rate of successful eye tracking largely because they performed post-calibration, since their protocol consisted of a single stimulus in a known position on an otherwise blank screen (Hagenaar et al., 2024). Because of the naturalistic scenes used for saliency analysis, post-calibration was not possible in our study.

### 4.5 Relationship of CVI classification to developmental (MSEL) and adaptive behavior (VABS-III) scores

Since there is no single gold standard to diagnose CVI in children with neurogenetic syndromes, we compared CVI classification by ophthalmologic examination, eye tracking, and functional vision questionnaires to results of the MSEL visual reception scale. Although the scale is intended as a measure of non-verbal cognition, it has been used to assess visual perception in children with a developmental age less than 4 years (Philip et al., 2023). We found that children in the 15q-CVI-DHS group had significantly lower MSEL visual reception developmental quotients compared to those in the 15q-CVI-LS group, supporting our method of classification. Children in the 15q-CVI-DHS group also had lower scores on other MSEL domains, including fine motor and verbal, although adaptive behavior scores on the VABS-III composite and domains did not differ between groups. The reason for the discrepancy is unclear, but could relate to compensatory behaviors in children with CVI that led to similar adaptive function despite greater delays in development.

### 4.6 Relationship of CVI classification to vision-specific quality of life (CVFQ) scores

The CVFQ results were also supportive of our classification. Children in the 15q-CVI-DHS group had worse vision-specific quality of life on the overall, competence, and personality scales, even after correcting for ophthalmologic comorbidities. However, the family impact subscale was not significantly different between groups. The questions on this subscale are related to time spent worrying about and treating a child’s visual diagnosis, and also parental concern about how a child’s eyes look. Since most of the children in the 15q-CVI-DHS group were not previously diagnosed with CVI, and CVI does not affect the appearance of the eyes, these items are not necessarily expected to be worse in children with CVI.

### 4.7 Ophthalmologic disorders other than CVI

With regard to rates of other ophthalmologic conditions in our 15q cohort, our findings are similar to prior literature in children with AS. To our knowledge, there are no case series describing ophthalmologic findings in children with Dup15q syndrome. With the exception of refractive errors, the rates of ophthalmologic disorders were similar in children with AS and Dup15q syndrome in our cohort. We diagnosed strabismus in 40% of 15q participants. In the literature, the frequency of strabismus in children with AS ranges from 36 to 75% (Galli et al., 2023; Dickinson et al., 1990; Michieletto et al., 2011; Buntinx et al., 1995; Micheletti et al., 2016; Khan et al., 2019). Amblyopia was diagnosed in 8% of our 15q participants. Rates of amblyopia are not consistently reported in the literature in children with AS, likely due to the challenges in assessing visual acuity in this population. Therefore, we cannot compare the frequency of amblyopia in our cohort to prior publications. However, previous investigations have noted high rates of iris and choroidal hypopigmentation in children with AS, ranging from 36 to 87% (Galli et al., 2023; Dickinson et al., 1990; Michieletto et al., 2011; Buntinx et al., 1995; Micheletti et al., 2016). We did not note pathologic hypopigmentation in any of our participants; however, we did not consider blue irides or fundus hypopigmentation without foveal hypoplasia (which are the characteristics of the phenotype reported in prior reports) to be pathologic since these findings are not expected to adversely affect visual function. We also did not find nystagmus in any of our patients. Nystagmus has previously been reported in 0 to 9% of children with AS (Galli et al., 2023; Dickinson et al., 1990; Michieletto et al., 2011; Micheletti et al., 2016).

In our cohort, we found that 60% of children with AS had a significant refractive error, significantly higher than 5% of children with Dup15q syndrome. Refractive errors in children with Dup15q syndrome have not been well-described; however, several case series have reported a high frequency of refractive errors (up to 97%) in children with Angelman syndrome (Dickinson et al., 1990; Michieletto et al., 2011; Micheletti et al., 2016). Prior studies generally have not distinguished between significant and non-significant refractive errors. The most common refractive errors in previous reports are hyperopia and astigmatism; the threshold for reporting was either not disclosed or >1 diopter. Symmetric low hyperopia in children typically does not require correction if accommodation is intact and the child is not esotropic (Hutchinson et al., 2023); therefore, prior studies of children with AS have likely overestimated the percentage of participants with significant refractive errors. In our study, we only reported refractive errors if they were significant and spectacle correction was recommended based on the AAO Preferred Practice Pattern (Hutchinson et al., 2023). Despite this, the rate of refractive errors in children with AS was quite high in our study compared to the general pediatric population. In contrast, the rate of significant refractive errors in children with Dup15q syndrome (5%) was not higher than population-based pediatric studies (Wen et al., 2013; Multi-Ethnic Pediatric Eye Disease Study G, 2010), although direct comparisons are not possible since prevalence of refractive errors varies widely by age as well as race and ethnicity.

### 4.8 Limitations

Our study must be interpreted in light of its limitations. A major limitation is that we combined children with AS and Dup15q syndrome to maximize sample size for analysis. Although the phenotypes of these syndromes overlap and we found no significant differences between the groups on demographic and clinical characteristics (except for rates of significant refractive errors), there are likely other differences between the groups that were not captured by our analyses or not detected due to the small sample size (particularly with regard to the Angelman syndrome group). Furthermore, we did not perform subgroup analysis based on genetic subtype, other than identifying a higher rate of 15q-CVI-DHS classification in the Dup15q idic subtype compared to the interstitial subtype. Further analysis of Dup15q and AS genetic subtypes was not performed since there were only 3 children with the Dup15q interstitial subtype and 5 children total with AS (with an unknown genetic subtype in 2).

Another important limitation is that we did not perform autism assessments. Prior studies have reported that up to 100% of children with Dup15q syndrome meet criteria for autism spectrum disorder (ASD) diagnosis (DiStefano et al., 2016), although the profile differs from children with non-syndromic autism. In Angelman syndrome, the association with ASD is less clear, with a phenotype that suggests social communication challenges in the setting of increased drive for social engagement (Pelc et al., 2008). Prior eye tracking studies have reported decreased fixations to social stimuli in children with ASD and those with Angelman syndrome (Hong et al., 2017; Falck-Ytter et al., 2013), which could potentially account for some of the abnormal saliency findings in our 15q-CVI-DHS group. For example, decreased fixations to human faces could be due to decreased visual social attention rather than challenges with visual processing. However, the abnormalities of visual attention on SET-CVI in the 15q-CVI-DHS group are not limited to social stimuli but extend to other visual characteristics requiring higher level processing to interpret, such as visual complexity, two-dimensional depictions of depth, and still depictions of motion. Thus, it is unlikely that comorbid ASD can account for all of the eye tracking abnormalities detected in the 15q-CVI-DHS children. Future studies are needed to compare eye tracking findings in 15q children with CVI, ASD, and both CVI and ASD.

Other limitations include the small sample size overall and potentially limited generalizability, since our participants were research volunteers from a clinic at a tertiary care children’s hospital. Due to the small sample size and exploratory nature of our analyses, correction for multiple comparisons was not performed. This decision was made in order to prevent type II errors during this hypothesis-generating phase of research. However, this increases the risk of type I errors, and borderline associations should be interpreted with caution. Furthermore, there was a wide age range among our participants, although we adjusted for age in our comparisons of eye tracking measures and vision-specific quality of life. We also acknowledge that independent validation of our technique in distinct cohorts of children with Angelman and Dup15q syndrome is needed. We used SET-CVI to identify children with “high suspicion” of CVI and justified this label by comparing CVI suspects to previously published cohorts of children with definite CVI and typically developing controls. Future studies could further validate SET-CVI by comparing eye tracking findings to results of functional vision assessments or standardized neuropsychological tests of visual perception in the subgroup of children with 15q disorders who have sufficient language and cognitive skills to perform these tests. CVI suspects were identified by eye tracking (which is not fully validated) and questionnaires (which are subjective). In contrast, CVI was diagnosed using established and objective clinical criteria. We grouped children with suspected and diagnosed CVI into a single category for pragmatic reasons due to small sample size, but the groups should not be considered equivalent. Children with CVI diagnosis may have more severe symptoms, and grouping them together with CVI suspects could have reduced the strength of some of the associations with developmental, adaptive behavior, and quality of life scores. Finally, limitations of eye tracking studies in general include special considerations for children with nystagmus and strabismus (those with high-amplitude nystagmus must be excluded, and those with strabismus must be analyzed using the fixating eye), as well as a minimum level of visual acuity required to view images on a computer monitor and a minimum degree of attention required to perform calibration. Despite this, as evidenced by our study, we are able to perform eye tracking successfully in the majority of children with neurogenetic conditions and severe developmental delays.

## 5 Conclusion

In conclusion, we report that children with Angelman and Dup15q syndromes have a high rate of diagnosis of CVI or CVI suspect. Additionally, our data suggest that eye tracking may identify CVI features in nearly half of 15q children who are ultimately diagnosed or suspected of having this condition, suggesting that eye tracking is a useful ancillary test in this population. Our method of classifying children as having a diagnosis of CVI or CVI suspect is supported by developmental and quality of life assessments. Borderline associations between CVI status and development in verbal and fine motor domains suggest that future studies of interventions for CVI could assess developmental progress in non-visual domains as a secondary outcome. Future research is also needed to validate our techniques in a larger and more diverse cohort of children with Angelman and Dup15q syndromes as well as other forms of significant intellectual disability, which will also enable more detailed phenotyping by genetic subtype.

## Data Availability

The raw data supporting the conclusions of this article will be made available by the authors, without undue reservation.

## Footnotes

1
https://biit.cs.ut.ee/clustvis/

## References

[ref1] Abbott M. Angione K. Stringfellow M. Malik K. Saenz M. McCourt E. . (2025). Cortical visual impairment across a range of neurodevelopmental disorders: clinical characterization, diagnostic tool evaluation, and association with developmental outcomes. J. Child Neurol. 41, 470–477. doi: 10.1177/08830738251361698, 40767165 PMC12360487

[ref2] Al Ageeli E. Drunat S. Delanoe C. Perrin L. Baumann C. Capri Y. . (2014). Duplication of the 15q11-q13 region: clinical and genetic study of 30 new cases. Eur. J. Med. Genet. 57, 5–14. doi: 10.1016/j.ejmg.2013.10.008, 24239951

[ref3] Avramidis K. Chang M. Y. Sharma R. Borchert M. S. Narayanan S. Evaluating atypical gaze patterns through vision models: the case of cortical visual impairment. 46th Annual International Conference of the IEEE Engineering in Medicine and Biology Society (EMBC); (2024) Orlando, FL: IEEE (Institute for Electrical and Electronics Engineers) 1–5. 10.1109/EMBC53108.2024.10782199 40039116

[ref4] Ben Itzhak N. Vancleef K. Franki I. Laenen A. Wagemans J. Ortibus E. (2020). Visuoperceptual profiles of children using the Flemish cerebral visual impairment questionnaire. Dev. Med. Child Neurol. 62, 969–976. doi: 10.1111/dmcn.14448, 31889310

[ref5] Birch E. E. Cheng C. S. Felius J. (2007). Validity and Reliability of the Children's Visual Function Questionnaire (CVFQ). J. AAPOS 11, 473–479. doi: 10.1016/j.jaapos.2007.02.011 PMC2063430 17512228

[ref6] Bisba M. Malamaki C. Constantoulakis P. Vittas S. (2024). Chromosome 15q11-q13 duplication syndrome: a review of the literature and 14 new cases. Genes 15:1304. doi: 10.3390/genes15101304, 39457428 PMC11507414

[ref7] Buffenn A. N. (2021). The impact of strabismus on psychosocial health and quality of life: a systematic review. Surv. Ophthalmol. 66, 1051–1064. doi: 10.1016/j.survophthal.2021.03.005, 33773997

[ref8] Buntinx I. M. Hennekam R. C. Brouwer O. F. Stroink H. Beuten J. Mangelschots K. . (1995). Clinical profile of Angelman syndrome at different ages. Am. J. Med. Genet. 56, 176–183. doi: 10.1002/ajmg.1320560213, 7625442

[ref9] Chandna A. Ghahghaei S. Foster S. Kumar R. (2021). Higher visual function deficits in children with cerebral visual impairment and Good visual acuity. Front. Hum. Neurosci. 15:711873. doi: 10.3389/fnhum.2021.711873, 34867236 PMC8636735

[ref10] Chang M. Y. Borchert M. S. (2020). Advances in the evaluation and management of cortical/cerebral visual impairment in children. Surv. Ophthalmol. 65, 708–724. doi: 10.1016/j.survophthal.2020.03.001, 32199940

[ref11] Chang M. Y. Borchert M. S. (2021a). Methods of visual assessment in children with cortical visual impairment. Curr. Opin. Neurol. 34, 89–96. doi: 10.1097/WCO.0000000000000877, 33230032

[ref12] Chang M. Y. Borchert M. S. (2021b). Validity and reliability of eye tracking for visual acuity assessment in children with cortical visual impairment. J. AAPOS 25, 334.e1–334.e5. doi: 10.1016/j.jaapos.2021.07.008, 34687876

[ref13] Chang M. Y. Borchert M. S. (2024). Comparison of eye tracking and teller acuity cards for visual acuity assessment in pediatric cortical/cerebral visual impairment. Am. J. Ophthalmol. 260, 115–121. doi: 10.1016/j.ajo.2023.11.024, 38065248 PMC10981560

[ref14] Chang M. Y. Borchert M. S. (2025). Cerebral/cortical visual impairment classification and categorization using eye tracking measures of oculomotor function. Ophthalmol. Sci. 5:100728. doi: 10.1016/j.xops.2025.100728, 40151356 PMC11946759

[ref15] Chang M. Merabet L. Group CW (2024). Special commentary: cerebral/cortical visual impairment working definition: a report from the National Institutes of Health CVI workshop. Ophthalmology 131, 1359–1365. doi: 10.1016/j.ophtha.2024.09.017, 39572128 PMC11588029

[ref16] Chang M. Y. Reid M. W. Roman Lantzy C. A. Contractor D. Granger K. O’Neil S. H. . (2026). Validity and reliability of the CVI range for clinical research (CVI range-CR): baseline and one-year results. Ophthalmol. Sci. 6:101233. doi: 10.1016/j.xops.2026.101233 PMC13273872 42317780

[ref17] Chang M. Roman-Lantzy C. O'Neil S. H. Reid M. W. Borchert M. S. (2022). Validity and reliability of CVI range assessment for clinical research (CVI range-CR): a longitudinal cohort study. BMJ Open Ophthalmol. 7, 1–8. doi: 10.1136/bmjophth-2022-001144, 42317780

[ref18] Cohen D. Martel C. Wilson A. Dechambre N. Amy C. Duverger L. . (2007). Brief report: visual-spatial deficit in a 16-year-old girl with maternally derived duplication of proximal 15q. J. Autism Dev. Disord. 37, 1585–1591. doi: 10.1007/s10803-006-0228-5, 17006777

[ref19] Colarusso R. P. Hammill D. D. (1972). Motor-free visual Perceptual test. Novato, CA: Academic Therapy Publications.

[ref20] Dickinson A. J. Fielder A. R. Young I. D. Duckett D. P. (1990). Ocular findings in Angelman's (happy puppet) syndrome. Ophthalmic Paediatr. Genet. 11, 1–6. doi: 10.3109/13816819009012942, 2348977

[ref21] DiStefano C. Gulsrud A. Huberty S. Kasari C. Cook E. Reiter L. T. . (2016). Identification of a distinct developmental and behavioral profile in children with Dup15q syndrome. J. Neurodev. Disord. 8:19. doi: 10.1186/s11689-016-9152-y, 27158270 PMC4858912

[ref22] DiStefano C. Sadhwani A. Wheeler A. C. (2020a). Comprehensive assessment of individuals with significant levels of intellectual disability: challenges, strategies, and future directions. Am. J. Intellect. Dev. Disabil. 125, 434–448. doi: 10.1352/1944-7558-125.6.434, 33211812

[ref23] DiStefano C. Wilson R. B. Hyde C. Cook E. H. Thibert R. L. Reiter L. T. . (2020b). Behavioral characterization of dup15q syndrome: toward meaningful endpoints for clinical trials. Am. J. Med. Genet. A 182, 71–84. doi: 10.1002/ajmg.a.61385, 31654560 PMC7334030

[ref24] Drover J. R. Wyatt L. M. Stager D. R. Birch E. E. (2009). The teller acuity cards are effective in detecting amblyopia. Optom. Vis. Sci. 86, 755–759. doi: 10.1097/OPX.0b013e3181a523a3, 19390474 PMC2869287

[ref25] Duis J. Nespeca M. Summers J. Bird L. Bindels-de Heus K. Valstar M. J. . (2022). A multidisciplinary approach and consensus statement to establish standards of care for Angelman syndrome. Mol. Genet. Genomic Med. 10:e1843. doi: 10.1002/mgg3.1843, 35150089 PMC8922964

[ref26] Falck-Ytter T. Bolte S. Gredeback G. (2013). Eye tracking in early autism research. J. Neurodev. Disord. 5:28. doi: 10.1186/1866-1955-5-28, 24069955 PMC3849191

[ref27] Fasaludeen A. Menon R. N. Jose M. Kumar A. A. Manisha K. Y. Radhakrishnan A. . (2026). Developmental and epileptic encephalopathy due to cyclin-dependent kinase-like 5 deficiency: a single-center experience across sex differences. Pediatr. Neurol. 177, 4–18. doi: 10.1016/j.pediatrneurol.2026.01.001, 41619470

[ref28] Galli J. Loi E. Strobio C. Micheletti S. Martelli P. Merabet L. B. . (2023). Neurovisual profile in children affected by Angelman syndrome. Brain Dev. 45, 117–125. doi: 10.1016/j.braindev.2022.10.003, 36344336

[ref29] Gorrie F. Goodall K. Rush R. Ravenscroft J. (2019). Towards population screening for cerebral visual impairment: validity of the five questions and the CVI questionnaire. PLoS One 14:e0214290. doi: 10.1371/journal.pone.0214290, 30913240 PMC6435113

[ref30] Hagenaar D. A. Bindels-de Heus K. van Gils M. M. van den Berg L. Ten Hoopen L. W. Affourtit P. . (2024). Outcome measures in Angelman syndrome. J. Neurodev. Disord. 16:6. doi: 10.1186/s11689-024-09516-1 PMC10905876 38429713

[ref31] Handa S. Saffari S. E. Borchert M. (2018). Factors associated with lack of vision improvement in children with cortical visual impairment. J. Neuroophthalmol. 38, 429–433. doi: 10.1097/WNO.0000000000000610, 29232345

[ref32] Hokken M. J. Geldof C. J. A. Stuit S. M. van der Zee Y. J. Moskalenko V. W. Escudero P. . (2025a). CVI questionnaires for preschool children: towards early screening of visual difficulties in daily life. Appl. Neuropsychol. Child, 1–11. doi: 10.1080/21622965.2025.2531422, 40674547

[ref33] Hokken M. J. Krabbendam E. van der Zee Y. J. Kooiker M. J. G. (2023). Visual selective attention and visual search performance in children with CVI, ADHD, and dyslexia: a scoping review. Child Neuropsychol. 29, 357–390. doi: 10.1080/09297049.2022.2057940, 35440276

[ref34] Hokken M. J. Stein N. Pereira R. R. Rours I. G. I. J. G. Frens M. A. van der Steen J. . (2024). Eyes on CVI: eye movements unveil distinct visual search patterns in cerebral visual impairment compared to ADHD, dyslexia, and neurotypical children. Res. Dev. Disabil. 151:104767. doi: 10.1016/j.ridd.2024.104767, 38861794

[ref35] Hokken M. J. van der Zee Y. J. van der Geest J. N. Kooiker M. J. G. (2025b). Parent-reported problems in children with cerebral visual impairment: improving the discriminative ability from ADHD and dyslexia using screening inventories. Neuropsychol. Rehabil. 35, 54–74. doi: 10.1080/09602011.2024.2328875, 38502713

[ref36] Hong M. P. Guilfoyle J. L. Mooney L. N. Wink L. K. Pedapati E. V. Shaffer R. C. . (2017). Eye gaze and pupillary response in Angelman syndrome. Res. Dev. Disabil. 68, 88–94. doi: 10.1016/j.ridd.2017.06.011, 28750207 PMC7169996

[ref37] Huo R. Burden S. K. Hoyt C. S. Good W. V. (1999). Chronic cortical visual impairment in children: aetiology, prognosis, and associated neurological deficits. Br. J. Ophthalmol. 83, 670–675. doi: 10.1136/bjo.83.6.670, 10340973 PMC1723072

[ref38] Hutchinson A. K. Morse C. L. Hercinovic A. Cruz O. A. Sprunger D. T. Repka M. X. . (2023). Pediatric eye evaluations preferred practice pattern. Ophthalmology 130, P222–P270. doi: 10.1016/j.ophtha.2022.10.030, 36543602 PMC10680450

[ref39] Khan N. Cabo R. Tan W. H. Tayag R. Bird L. M. (2019). Healthcare burden among individuals with Angelman syndrome: findings from the Angelman syndrome natural history study. Mol. Genet. Genomic Med. 7:e00734. doi: 10.1002/mgg3.734, 31090212 PMC6625091

[ref40] Kooiker M. J. van der Steen J. Pel J. J. (2016). Development of salience-driven and visually-guided eye movement responses. J. Vis. 16:18. doi: 10.1167/16.5.18 26998802

[ref41] Kooiker M. J. G. Verbunt H. J. M. van der Steen J. Pel J. J. M. (2018). Combining visual sensory functions and visuospatial orienting functions in children with visual pathology: a longitudinal study. Brain Dev. 41, 135–149. doi: 10.1016/j.braindev.2018.09.006, 30301589

[ref42] Leske D. A. Hatt S. R. Wernimont S. M. Castaneda Y. S. Cheng-Patel C. S. Liebermann L. . (2021). Quality of life and functional vision across pediatric eye conditions assessed using the PedEyeQ. J. AAPOS 25, 23.e1–e5. doi: 10.1016/j.jaapos.2020.10.010, 33601046 PMC8169574

[ref43] Macintyre-Beon C. Young D. Calvert J. Ibrahim H. Dutton G. N. Bowman R. (2012). Reliability of a question inventory for structured history taking in children with cerebral visual impairment. Eye 26:1393. doi: 10.1038/eye.2012.154, 22863818 PMC3470063

[ref44] Manley C. E. Bennett C. R. Merabet L. B. (2022). Assessing higher-order visual processing in cerebral visual impairment using naturalistic virtual-reality-based visual search tasks. Children 9:1114. doi: 10.3390/children9081114, 35892617 PMC9331719

[ref45] Manley C. E. Walter K. Micheletti S. Tietjen M. Cantillon E. Fazzi E. M. . (2023). Object identification in cerebral visual impairment characterized by gaze behavior and image saliency analysis. Brain Dev. 45, 432–444. doi: 10.1016/j.braindev.2023.05.001, 37188548 PMC10524860

[ref46] Martin N. A. (2017). Test of Visual Perceptual Skills-4th edition (TVPS-4). Novato, CA: Academic Therapy Publications.

[ref47] Matsunaga K. Avramidis K. Borchert M. S. Narayanan S. Chang M. Y. (2024). Method for assessing visual saliency in children with cerebral/cortical visual impairment using generative artificial intelligence. Front. Hum. Neurosci. 18:1506286. doi: 10.3389/fnhum.2024.1506286, 39897082 PMC11782282

[ref48] Matsunaga K. Avramidis K. Borchert M. Narayanan S. Chang M. Y. (2026). Generative artificial intelligence-enabled saliency analysis of eye tracking in cerebral/cortical visual impairment (SET-CVI). Ophthalmol. Sci. 6:101081. doi: 10.1016/j.xops.2026.101081, 41743165 PMC12930050

[ref49] Mayer D. L. Beiser A. S. Warner A. F. Pratt E. M. Raye K. N. Lang J. M. (1995). Monocular acuity norms for the teller acuity cards between ages one month and four years. Invest. Ophthalmol. Vis. Sci. 36, 671–685. 7890497

[ref50] Metsalu T. Vilo J. (2015). ClustVis: a web tool for visualizing clustering of multivariate data using principal component analysis and heatmap. Nucleic Acids Res. 43, W566–W570. doi: 10.1093/nar/gkv468, 25969447 PMC4489295

[ref51] Micheletti S. Palestra F. Martelli P. Accorsi P. Galli J. Giordano L. . (2016). Neurodevelopmental profile in Angelman syndrome: more than low intelligence quotient. Ital. J. Pediatr. 42:91. doi: 10.1186/s13052-016-0301-4, 27769316 PMC5073425

[ref52] Michieletto P. Bonanni P. Pensiero S. (2011). Ophthalmic findings in Angelman syndrome. J. AAPOS 15, 158–161. doi: 10.1016/j.jaapos.2010.12.013, 21596294

[ref53] Mullen E. M. (1995). Mullen Scales of Early Learning. Circle Pines, MN: American Guidance Services Inc.

[ref54] Multi-Ethnic Pediatric Eye Disease Study G (2010). Prevalence of myopia and hyperopia in 6- to 72-month-old african american and Hispanic children: the multi-ethnic pediatric eye disease study. Ophthalmology 117, 140–147.e3. doi: 10.1016/j.ophtha.2009.06.009, 19926137 PMC2815146

[ref55] Musleh M. Green A. Mankowska A. Viner C. Pilling R. (2024). Spectrum of visual dysfunction detected by a novel testing protocol within a special school eye care service. Br. Ir. Orthopt. J. 20, 219–225. doi: 10.22599/bioj.391, 39552718 PMC11568808

[ref56] Pelc K. Cheron G. Dan B. (2008). Behavior and neuropsychiatric manifestations in Angelman syndrome. Neuropsychiatr. Dis. Treat. 4, 577–584. doi: 10.2147/NDT.S2749, 18830393 PMC2526368

[ref57] Philip J. Huurneman B. Jansonius N. M. Cillessen A. H. N. Boonstra F. N. (2023). Childhood cerebral visual impairment subtype classification based on an extensive versus a limited test battery. Front. Neurosci. 17:1266201. doi: 10.3389/fnins.2023.1266201, 37954874 PMC10637406

[ref58] Shic F. Naples A. J. Barney E. C. Chang S. A. Li B. McAllister T. . (2022). The autism biomarkers consortium for clinical trials: evaluation of a battery of candidate eye-tracking biomarkers for use in autism clinical trials. Mol. Autism. 13:15. doi: 10.1186/s13229-021-00482-2, 35313957 PMC10124777

[ref59] Sparrow S. S. Cicchetti D. V. Saulnier C. A. (2016). Vineland Adaptive Behavior Scales, Third Edition. San Antonio, TX: Pearson.

[ref60] Van Splunder J. Stilma J. S. Evenhuis H. M. (2003). Visual performance in specific syndromes associated with intellectual disability. Eur. J. Ophthalmol. 13, 566–574. doi: 10.1177/112067210301300610, 12948316

[ref61] Walter K. Manley C. E. Bex P. J. Merabet L. B. (2024). Visual search patterns during exploration of naturalistic scenes are driven by saliency cues in individuals with cerebral visual impairment. Sci. Rep. 14:3074. doi: 10.1038/s41598-024-53642-8, 38321069 PMC10847433

[ref62] Wen G. Tarczy-Hornoch K. McKean-Cowdin R. Cotter S. A. Borchert M. Lin J. . (2013). Prevalence of myopia, hyperopia, and astigmatism in non-Hispanic white and Asian children: multi-ethnic pediatric eye disease study. Ophthalmology 120, 2109–2116. doi: 10.1016/j.ophtha.2013.06.039, 23953098 PMC3902090

[ref63] Williams C. Pease A. Warnes P. Harrison S. Pilon F. Hyvarinen L. . (2021). Cerebral visual impairment-related vision problems in primary school children: a cross-sectional survey. Dev. Med. Child Neurol. 63, 683–689. doi: 10.1111/dmcn.14819, 33533021

[ref64] Zatta M. C. Williems I. (2024). Development of a new assessment for cerebral or cortical visual impairment: the Perkins CVI protocol. J. Vis. Impair. Blind. 118, 442–447. doi: 10.1177/0145482X241297989

