# Supplementary material for: Vision In SyndrOmic Neurodevelopmental disorders-15q (VISiON-15q) study: eye tracking to supplement clinical examination for detection of cerebral/cortical visual impairment in children with Angelman and Duplication of 15q Syndromes
Source: Front Hum Neurosci. 2026 Sep 29;20:1953706. doi: 10.3389/fnhum.2026.1953706 (PMC13658069; doi:10.3389/fnhum.2026.1953706)
Supplement: Supplementary file 1 [file Table_1.DOCX]

Supplemental Table 1. Visual Behavior Scale (VBS), 6-level scale of visual acuity based on visual behavior.

| 1 | Light perception only |
| --- | --- |
| 2 | Occasional fixation on large objects, faces, or movement |
| 3 | Occasional fixation on small objects or reliable fixation on large objects (4-inch lighted toy at 1 foot) or faces |
| 4 | Reliable fixation on small objects (2-inch toy at 1 foot) |
| 5 | Reliable fixation and pursuit of small objects (2-inch toy at 1 foot) |
| 6 | Reliable fixation and pursuit of smallest objects (1-inch toy at least 2 feet away) |
