# Supplementary material for: Vision In SyndrOmic Neurodevelopmental disorders-15q (VISiON-15q) study: eye tracking to supplement clinical examination for detection of cerebral/cortical visual impairment in children with Angelman and Duplication of 15q Syndromes
Source: Front Hum Neurosci. 2026 Sep 29;20:1953706. doi: 10.3389/fnhum.2026.1953706 (PMC13658069; doi:10.3389/fnhum.2026.1953706)
Supplement: Supplementary file 2 [file Supplementary_file_1.zip › Supplementary Figures/Figure 1.DOCX]

Supplemental Figure 1. Method to create saliency maps to 15 lower- and higher-level visual characteristics. Saliency maps for lower-level visual characteristics were created using pixel values, and saliency maps for higher-level visual characteristics were created using a generative artificial intelligence (AI) model. Used with permission from: Matsunaga K, Avramidis K, Borchert M, et al. Generative Artificial Intelligence-Enabled Saliency Analysis of Eye Tracking in Cerebral/Cortical Visual Impairment (SET-CVI). Ophthalmol Sci. 2026;6(4):101081.
