# Supplementary material for: Vision In SyndrOmic Neurodevelopmental disorders-15q (VISiON-15q) study: eye tracking to supplement clinical examination for detection of cerebral/cortical visual impairment in children with Angelman and Duplication of 15q Syndromes
Source: Front Hum Neurosci. 2026 Sep 29;20:1953706. doi: 10.3389/fnhum.2026.1953706 (PMC13658069; doi:10.3389/fnhum.2026.1953706)
Supplement: Supplementary file 2 [file Supplementary_file_1.zip › Supplementary Figures/Figure 2.DOCX]

Supplemental Figure 2. Method to calculate fixation saliency values. Used with permission from: Matsunaga K, Avramidis K, Borchert MS, et al. Method for assessing visual saliency in children with cerebral/cortical visual impairment using generative artificial intelligence. Front Hum Neurosci. 2024;18:1506286.
